# Sex differences in the circadian profiles of melatonin and cortisol in plasma and urine matrices under constant routine conditions

**DOI:** 10.3109/07420528.2015.1112396

**Published:** 2016-01-05

**Authors:** Pippa J. Gunn, Benita Middleton, Sarah K. Davies, Victoria L. Revell, Debra J. Skene

**Affiliations:** ^a^ Chronobiology, Faculty of Health and Medical Sciences, University of Surrey, Guildford, United Kingdom; ^b^ Radcliffe Department of Medicine, University of Oxford, Level 6, West Wing, John Radcliffe Hospital, Headington, Oxford, United Kingdom; ^c^ Faculty of Medicine, Imperial College London, London, United Kingdom

**Keywords:** Sex differences, melatonin, cortisol, 6-sulfatoxymelatonin, constant routine, circadian rhythms, human

## Abstract

Conflicting evidence exists as to whether there are differences between males and females in circadian timing. The aim of the current study was to assess whether sex differences are present in the circadian regulation of melatonin and cortisol in plasma and urine matrices during a constant routine protocol. Thirty-two healthy individuals (16 females taking the oral contraceptive pill (OCP)), aged 23.8 ± 3.7 (mean ± SD) years, participated. Blood (hourly) and urine (4-hourly) samples were collected for measurement of plasma melatonin and cortisol, and urinary 6-sulfatoxymelatonin (aMT6s) and cortisol, respectively. Data from 28 individuals (14 females) showed no significant differences in the timing of plasma and urinary circadian phase markers between sexes. Females, however, exhibited significantly greater levels of plasma melatonin and cortisol than males (AUC melatonin: 937 ± 104 (mean ± SEM) vs. 642 ± 47 pg/ml.h; AUC cortisol: 13581 ± 1313 vs. 7340 ± 368 mmol/L.h). Females also exhibited a significantly higher amplitude rhythm in both hormones (melatonin: 43.8 ± 5.8 vs. 29.9 ± 2.3 pg/ml; cortisol: 241.7 ± 23.1 vs. 161.8 ± 15.9 mmol/L). Males excreted significantly more urinary cortisol than females during the CR (519.5 ± 63.8 vs. 349.2 ± 39.3 mol) but aMT6s levels did not differ between sexes. It was not possible to distinguish whether the elevated plasma melatonin and cortisol levels observed in females resulted from innate sex differences or the OCP affecting the synthetic and metabolic pathways of these hormones. The fact that the sex differences observed in total plasma concentrations for melatonin and cortisol were not reproduced in the urinary markers challenges their use as a proxy for plasma levels in circadian research, especially in OCP users.

## Introduction

Diurnal rhythms observed in human physiology and behaviour result from an interaction between endogenous signals, originating in the circadian timing system, and external signals from the physical environment, such as light-dark cycles, activity or food. Use of constant routine (CR) protocols is an accepted method in human research to remove or minimise external time cues and allow rhythms driven only by endogenous circadian stimuli to be identified (Duffy and Dijk, 2002). Melatonin and cortisol are two well-characterised rhythmic hormones which are driven by the master circadian clock, located in the suprachiasmatic nuclei (SCN) of the anterior hypothalamus. In entrained conditions, melatonin levels rise in the evening hours, peak in the early hours of the morning, and return to basal levels soon after waking (Arendt and Skene, 2005). Cortisol begins to increase in the early hours, peaks after wake time and decreases throughout the rest of the day (Debono et al., 2009; Weitzman et al., 1971).

**Figure 1.**
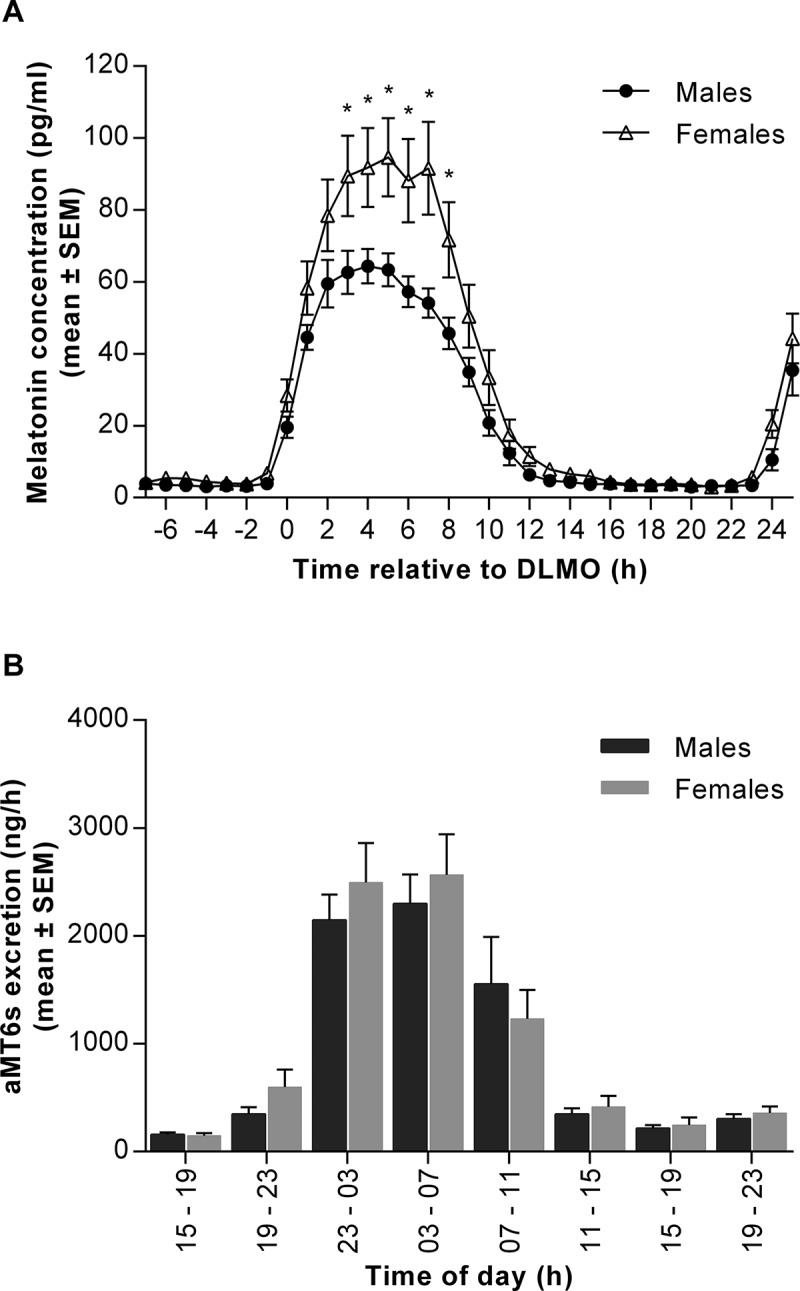
Melatonin production and excretion of 6-sulfatoxymelatonin (aMT6s) in males and females measured over 32 hours during a constant routine (CR) protocol. (**A**) Plasma melatonin concentrations measured at hourly intervals in males (black circles) and females (open triangles) over 32 hours. Data are plotted relative to each person’s DLMOn_25%_, designated to occur at 0 h. *p < 0.05 between sexes. Two-way ANOVA with Sidak’s multiple comparisons test, time effect *p* < 0.0001; sex effect *p* < 0.05; time x sex interaction *p* < 0.0001. (**B**) Urinary aMT6s excretion expressed per hour from eight timed collections in males (dark bars) and females (light bars). Two-way ANOVA with Sidak’s multiple comparisons test, time effect *p* < 0.0001; sex effect and time x sex interaction not significant (*p* > 0.05). Data are mean ± SEM, *n* = 14 for each group.

As a result of being under the control of the SCN oscillator, melatonin is the marker of choice for circadian phase estimation, whereas cortisol, due to being influenced by external factors and its intrinsic variability, is less commonly used (Klerman et al., 2002). Dim light melatonin onset (DLMO) is a highly utilised marker of circadian phase, representing the time at which melatonin levels begin to rise (Lewy and Sack, 1989). When investigated in both field and laboratory settings, DLMO has been found to occur significantly earlier in women than men (Cain et al., 2010; Mongrain et al., 2004; Van Reen et al., 2013). In addition, the phase angle between DLMO and habitual bedtime has been found to be shorter in women (Burgess and Eastman, 2005). However, the field settings or limited control of laboratory conditions in many of these studies mean it is difficult to conclude whether these results arose due to differences in the endogenous circadian rhythm of melatonin or from the influence of other exogenous signals.

As well as differences in the timing of circadian phase markers, differences in circulating melatonin and cortisol concentrations have been observed between sexes. Although studies measuring a single sample of cortisol, which are of limited utility, have reported no sex differences (Kudielka et al., 2004; Seeman et al., 2001), the use of a morning and evening sample showed women had a higher cortisol level in the morning, but not the evening, compared to men (Larsson et al., 2009). By contrast, samples taken at least every 30 minutes across a 24-hour sampling period revealed a lower mean production of plasma cortisol in females (Van Cauter et al., 1996). Moreover, Cain et al. (2010) reported increased production of plasma melatonin in females during their CR study. In addition, evidence exists that the use of the oral contraceptive pill (OCP) may affect hormone levels, with reports that both melatonin and cortisol increase with the use of OCPs (Kostoglou-Athanassiou et al., 1998; Simunkova et al., 2008). However, these studies lacked high resolution sampling across 24 hours or more, thus not providing a detailed picture of melatonin and cortisol production.

In addition to measuring circulating levels of melatonin and cortisol, it is also possible to utilise their urinary metabolites as circadian phase markers. Melatonin is excreted as 6-sulfatoxymelatonin (aMT6s) in the urine and aMT6s is a well-established proxy measure of circulating melatonin and circadian timing, strongly correlating with plasma melatonin (Arendt et al., 1985; Kovacs et al., 2000). Cortisol is excreted in the urine as a number of metabolites along with free cortisol. Like aMT6s, the timing of urinary cortisol rhythms has been shown to be useful in tracking circadian rhythms in the blind (Hack et al., 2003; Lockley et al., 2000; Skene et al., 1999), with available research suggesting a moderate association between plasma and urinary measures (Jung et al., 2014). For both aMT6s and urinary cortisol there is some evidence of sex-dependent effects (Finken et al., 1999; Shamim et al., 2000; Wetterberg et al., 1999), although limited sampling points and unavailability of plasma levels for comparison make these sex differences difficult to interpret.

Previously, CR protocols have been under-utilised when examining sex differences in phase markers and have been limited by their use of plasma melatonin as a single circadian marker (Cain et al., 2010). The aim of this study was to investigate the timing and production of the circadian SCN clock-controlled hormones, melatonin and cortisol, in males and females in unmasked conditions using a CR protocol. The use of this protocol allowed any sex differences in the endogenous regulation of these hormones to be characterised. Additionally, through collection of urinary aMT6s and cortisol in the same individuals and during the same protocol, we aimed to examine whether sex differences existed in the metabolism and excretion of melatonin and cortisol, allowing a novel and comprehensive assessment of circadian markers in males and females during a CR protocol.

## Materials and methods

### Study

Thirty two healthy participants (16 females) aged between 19 and 33 years (23.7 ± 3.6 years; mean ± SD) took part in the study. Two participants (one female) were excluded from the analysis due to the need for them to take paracetamol during the in-laboratory period. One female participant was excluded as blood was drawn for only the first 24 hours of the study period. One male participant was excluded after being identified as an outlier (> ± 2 SD from the mean) in seven variables relating to melatonin production, timing and excretion. Therefore, samples from 28 participants (14 females) were included in the analysis (23.8 ± 3.7 years) (Table 1). The study was conducted between January and August 2012.

**Table 1.** Baseline characteristics and habitual sleep time (mean ± SEM) of male and female participants.

	Males (*n* = 14)	Females (*n* = 14)	*p* value
Age (years)	23.8 ± 1.1	23.7 ± 0.9	0.972^†^
Weight (kg)	76.8 ± 3.3	63.9 ± 2.2	0.003*
BMI (kg/m^2^)	23.8 ± 0.8	23.4 ± 0.6	0.688
Bedtime (dec.h)	23.82 ± 0.18	23.50 ± 0.16	0.188
Wake time (dec.h)	7.82 ± 0.18	7.50 ± 0.16	0.188
Horne–Östberg score	51.7 ± 1.8	53.4 ± 1.5	0.461

* Significant difference between sexes (*p* < 0.05). Independent *t-*tests, ^†^ indicates Mann-Whitney test used. Data are mean ± SEM.

All study procedures were conducted at the Surrey Clinical Research Centre (CRC). Participant eligibility was determined by medical and physical assessments, urine and blood analysis, and sleep and health questionnaires. All participants were non-smokers, free of existing medical conditions, prescription medication (excluding the OCP), and haematological abnormalities, and showed no evidence of drug or alcohol abuse. None had a history of shift work or travel across two or more time zones in the preceding month. All participants met defined criteria in four questionnaires: ≤ 5 in the Pittsburgh Sleep Quality Index, < 10 in the Beck Depression Inventory, < 10 in the Epworth Sleepiness Scale, and not extreme evening or morning types in the Horne–Östberg Questionnaire. Participants had a regular sleep-wake cycle with a bedtime between 22:00 and 01:00 h and wake time between 06:00 and 09:00 h with eight hours in bed. To be included all females had to be takingthe OCP for contraception and had to be in the active pill phase during the in-laboratory period. Oral contraceptives used were one of four formulations: 30 µg ethinylestradiol and 150 µg progestin (*n* = 10), 30 µg ethinylestradiol and 3000 µg progestin (*n* = 2), 35 µg ethinylestradiol and 250 µg progestin (*n* = 1) or 0 µg ethinylestradiol and 75 µg progestin (*n* = 1). None of the female participants had a history of menstrual irregularities prior to OCP use.

The study was approved by the University of Surrey Ethics Committee and conducted in accordance with the Declaration of Helsinki. The experimental protocol of the study conformed to international ethical standards (Portaluppi et al., 2010). Oral and written informed consent for the study was obtained from all participants before any study procedures were performed.

### In-laboratory session

The in-laboratory session was conducted at the Surrey CRC. Seven days prior to entering the CRC, participants were required to maintain a self-selected, habitual eight-hour sleep schedule (bedtime between 22:00 and 01:00 h) and receive at least 15 minutes of outdoor light in the first 90 minutes of waking. These procedures were to minimise any confounding sleep deprivation and to stabilise the circadian clock prior to the laboratory study. Compliance to this schedule was monitored by completion of a sleep and nap diary, calling a time-stamped voicemail upon waking and wearing an Actiwatch (Cambridge Neurotechnology Ltd., Cambridge, UK). For 72 hours prior to the in-laboratory session participants were asked to refrain from alcohol, exercise, bright light in the evening and caffeine.

The in-laboratory session was 68 hours in total, beginning at 16:00 h on Day 1 and ending at 12:00 h on Day 4. After an adaptation night, participants awoke at their habitual time on Day 2 into CR conditions of dim light (< 5 lux in the direction of gaze), maintaining a semi-recumbent posture, continual wakefulness and consuming hourly isocaloric snacks with 100 ml water. Participants had no knowledge of clock time during this period. The CR continued until 23:00 h on Day 3, and was followed by the opportunity for a recovery sleep.

Blood samples were drawn hourly from intravenous cannulae from 15:00 h on Day 2 until 23:00 h on Day 3. Samples were collected into lithium heparin tubes, separated by centrifugation and the plasma fraction stored at -20°C until analysis. All urine passed between habitual bedtime on Day 1 and 23:00 h on Day 3 was collected. The first collection period was 8 hours, between habitual bedtime and wake time, the second collection period covered habitual wake time to 11:00 h on Day 2, and all subsequent collection periods were four-hourly. Urine samples were frozen within 1 hour of the end of the collection interval and stored at -20°C until analysis.

Plasma melatonin and cortisol and urinary aMT6s and cortisol were measured by radioimmunoassay (Stockgrand Ltd., University of Surrey, UK) as previously described (Aldhous and Arendt, 1988; Fraser et al., 1983; Lockley et al., 2000; Riad-Fahmy et al., 1979). All samples from one participant were run in the same assay and measured in duplicate. The limit of detection for the plasma melatonin assay was 3.9 pg/ml and inter-assay coefficient of variations (CVs) were 22.2% at 8.2 pg/ml, 10.4% at 33.2 pg/ml, 9.3% at 88.8 pg/ml and 9.0% at 121.4 pg/ml. The limit of detection for the aMT6s assay was 0.4 ng/ml with inter-assay CVs of 9.2% at 2.9 ng/ml, 4.2% at 12.1 ng/ml and 2.3% at 21.1 ng/ml. The limit of detection for the plasma cortisol assay was 1.7 nmol/L and inter-assay CVs were 11.2% at 67.7 nmol/L, 11.0% at 520.0 nmol/L and 8.7% at 884.0 nmol/L. The limit of detection for the urinary cortisol assay was 2.2 nmol/L with inter-assay CVs of 12.7% at 84 nmol/L, 9.5% at 156.0 nmol/L and 16.8% at 736.8 nmol/L.

### Data analysis

In order to assess 24-hour rhythmicity of the hormones, cosinor analysis was carried out (Minors and Waterhouse, 1989). This analysis determined the amplitude, acrophase and significance of fit to a cosine curve for each individual hormone profile. For each participant, all 33 hourly plasma samples and all urine samples collected during the CR were included in the cosinor analysis for maximum validity of fitting.

To determine circadian phase, a number of markers were calculated. Dim light melatonin onset (DLMOn_25%_) and offset (DLMOff_25%_) were defined as the time at which plasma melatonin levels reached 25% of the maximum melatonin values (Davies et al., 2014; Sletten et al., 2009) on the evening on Day 2 and the morning of Day 3, respectively. The following phase angles were calculated: DLMOn_25%_ relative to bedtime and wake time (bedtime - DLMOn_25%_ and DLMOn_25%_ - wake time, respectively).

In order to investigate the relationship between plasma and urine concentrations of melatonin and aMT6s and cortisol, clearance estimates were calculated:

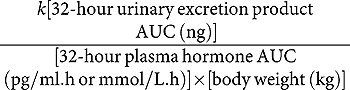

(where *k* = molecular weight of urinary excretion product (g/mol)/molecular weight of plasma hormone (g/mol))

### Statistical analysis

Statistical analyses were carried out using GraphPad Prism v6 (GraphPad Software, Inc., La Jolla, CA). AUC was determined for each plasma and urinary hormone across the entire protocol and, for plasma melatonin, additionally between DLMOn_25%_ and DLMOff_25%_. Data sets were first assessed for normality, with between sex differences in parametric and non-parametric variables determined by a two-tailed independent Student’s *t-*test and the Mann-Whitney *U*-test, respectively. Correlation analysis was performed using Pearson’s correlation coefficient, or Spearman’s rank correlation coefficient for non-parametric distributions. To assess sex differences across time points, a two-way ANOVA (factors: time and sex) was used with Sidak’s *post-hoc* tests on urine and DLMOn_25%_ corrected plasma values. Results refer to mean ± SEM and significance levels were set at *p* < 0.05.

## Results

Of the 28 participants analysed, males and females did not differ significantly in their age or BMI, although males had significantly higher body weight than the females (Table 1). Neither morningness-eveningness, as indicated by their Horne–Östberg score, nor habitual sleep times differed between sexes (bedtime range 22.50 to 01.00 dec.h in males vs. 22.50 to 00.50 dec.h in females, Table 1).

The amplitude of the melatonin rhythm during Night 2 of the CR differed significantly between men and women, with females exhibiting a higher peak concentration (Table 2). These differences in melatonin concentration were evident throughout the CR; the AUC calculated over 32 hours was significantly higher in women than men (Table 2), with significantly elevated levels in females between 3 and 8 hours after DLMOn_25%_ (Figure 1A). Season had no significant effect on the observed sex differences (sex, *p* < 0.05; season effect and sex x season interaction, *p* > 0.05).

**Table 2.** Sex differences in parameters relating to melatonin and 6-sulfatoxymelatonin timing, production and excretion.

	Males (*n* = 14)	Females (*n* = 14)	*p* value
Melatonin amplitude (pg/ml)	29.9 ± 2.3	43.8 ± 5.8	0.035*
32-hr melatonin AUC (pg/ml.h)	642 ± 47	937 ± 104	0.016*
DLMOn_25%_ (dec.h)	22.01 ± 0.25	21.7 ± 0.23	0.356
DLMOff_25%_ (dec.h)	8.23 ± 0.32	8.07 ± 0.26	0.698
DLMOn_25%_ to bedtime phase angle (h)	1.81 ± 0.24	1.8 ± 0.2	0.991
Wake time to DLMOn_25%_ phase angle (h)	14.13 ± 0.23	14.22 ± 0.19	0.766
DLMOff_25%_ to wake time phase angle (h)	-0.41 ± 0.29	-0.57 ± 0.27	0.690
Melatonin acrophase (dec.h)	3.36 ± 0.29	3.14 ± 0.21	0.547
32-hr aMT6s excretion (µg)	29.5 ± 2.7	32.2 ± 4.1	0.580
aMT6s acrophase (dec.h)	4.29 ± 0.33	3.72 ± 0.31	0.312^†^
Melatonin clearance (L/kg/h)	0.84 ± 0.05	0.78 ± 0.06	0.289

*Significant difference between sexes (*p* < 0.05). Independent *t-*tests, ^†^ndicates Mann-Whitney test used. Data are mean ± SEM. DLMOn_25%_, dim light melatonin onset; DLMOff_25%_, dim light melatonin offset; aMT6s, 6-sulfatoxymelatonin.

To investigate differences in phase timing between sexes, a number of markers were compared (Table 2). There were no significant differences identified between men and women in the timing of plasma DLMOn_25%_ or DLMOff_25%_, or the duration of melatonin production between these 25% thresholds (10.22 ± 0.22 h in males vs. 10.38 ± 0.30 h in females, *p* = 0.676). Likewise, there was no difference between males and females in the timing of the melatonin rhythm, with the clock time at which the acrophase occurred not differing significantly between groups (Table 2). The clock times of the DLMOn_25%_ and the cosinor acrophase were strongly correlated in males (*r* = 0.88, *p* < 0.0001), females (*r* = 0.69, *p* = 0.006) and the group as a whole (*r* = 0.80, *p* < 0.0001). The calculated phase angles for DLMOn_25%_ relative to bedtime and wake time, DLMOff_25%_ to wake time, and T_min_ to wake time did not differ significantly between groups (Table 2).

In contrast to the plasma melatonin data, there were no sex differences in the excretion of the urinary metabolite aMT6s over the 32-hour CR protocol (Table 2, Figure 1B). The discrepancy between plasma production and urinary excretion was not explained by a sex difference in the clearance rate, which was not significantly different between groups (Table 2). There was no evidence of an effect of sex on the timing of melatonin excretion, as indicated by similar aMT6s acrophases in men and women (Table 2). Levels of plasma melatonin and urinary aMT6s were highly correlated in both males (*r* = 0.85, *p* <0.0001), females (*r* = 0.79, *p* = 0.0007) and the group as a whole (*r* = 0.75, *p* < 0.001).

**Figure 2.**
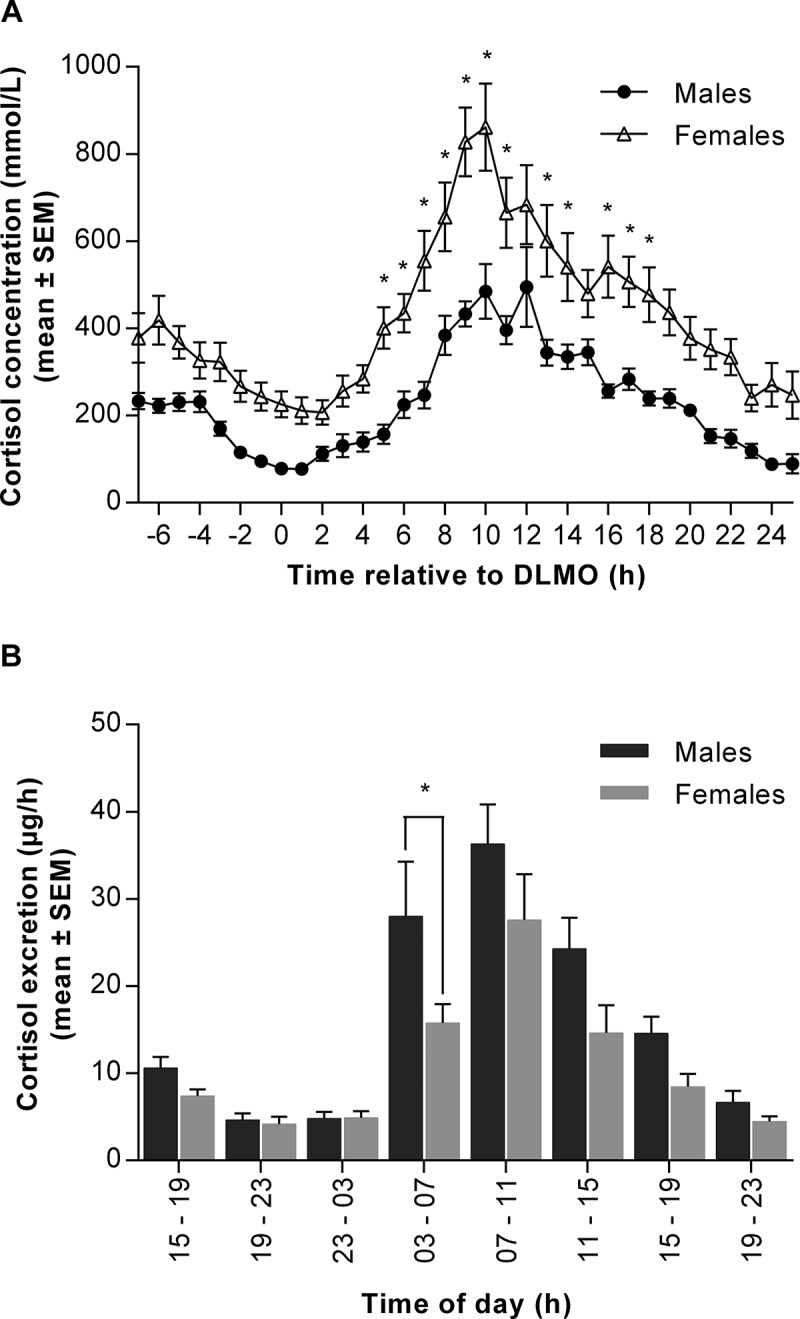
Cortisol production and excretion in males and females measured over 32 hours during a constant routine (CR) protocol. (**A**) Plasma cortisol concentration measured at hourly intervals in males (black circles) and females (open triangles) over 32 hours. Data are plotted relative to each person’s DLMOn_25%_, designated to occur at 0 h. *p < 0.05 between sexes at indicated time points. Two-way ANOVA with Sidak’s multiple comparisons test, time effect *p* < 0.0001; sex effect *p* < 0.0001; time x sex interaction *p* < 0.0001. (**B**) Urinary cortisol excretion expressed per hour from eight timed collections in males (dark bars) and females (light bars). *p < 0.05 between sexes at indicated time points. Two-way ANOVA with Sidak’s multiple comparisons test, time effect *p* < 0.0001; sex effect *p* < 0.05; time x sex interaction not significant (*p* > 0.05). Data are mean ± SEM, *n* = 14 for each group.

Plasma cortisol also showed significant differences between sexes (Table 3). Women produced more cortisol than men over the 32-hour CR protocol with a higher amplitude observed in the females. Season had no significant effect on the observed sex differences (sex, *p* < 0.001; season effect and sex x season interaction, *p* > 0.05). The difference between sexes was sustained throughout the protocol and statistically significant at 12 time points (5-18 hours after DLMO) (Figure 2A). These differences were particularly evident around the acrophase of cortisol production, the timing of which did not differ between males and females (Table 3). These findings were reversed in the urinary cortisol data, with males excreting significantly more cortisol than females at a higher clearance rate. However, the timing of the urinary cortisol rhythm, like the plasma data, did not differ between sexes (Table 3, Figure 2B). The difference between plasma and urinary levels of cortisol was additionally illustrated by an absence of correlation between plasma and urine concentrations in the whole group (*r* = -0.19, *p* = 0.342) and females (*r* = 0.13, *p* = 0.670), although there was a non-significant trend for a positive association in males (*r* = 0.48, *p* = 0.08).

**Table 3.** Sex differences in parameters relating to cortisol timing, production and excretion.

	Males (*n* = 14)	Females (*n* = 14)	*p* value
32-hr cortisol AUC (mmol/L.h)	7340 ± 368	13581 ± 1313	0.0001*
Cortisol amplitude (mmol/L)	161.8 ± 15.9	241.7 ± 23.1	0.019^a^*
Cortisol acrophase (dec.h)	10.04 ± 0.23	9.51 ± 0.23	0.079^a^
Urinary cortisol acrophase (dec.h)	9.12 ± 0.32	9.04 ± 0.40	0.855
32-hr urinary cortisol excretion (mol)	519.5 ± 63.8	349.2 ± 39.3	0.031^†^*
Cortisol clearance (L/kg/h)	0.92 ± 0.08	0.48 ± 0.09	0.0008*

*Significant difference between sexes (*p* < 0.05). Independent *t*-tests, ^†^ indicates Mann-Whitney test used. Data are mean ± SEM.

**Figure 3.**
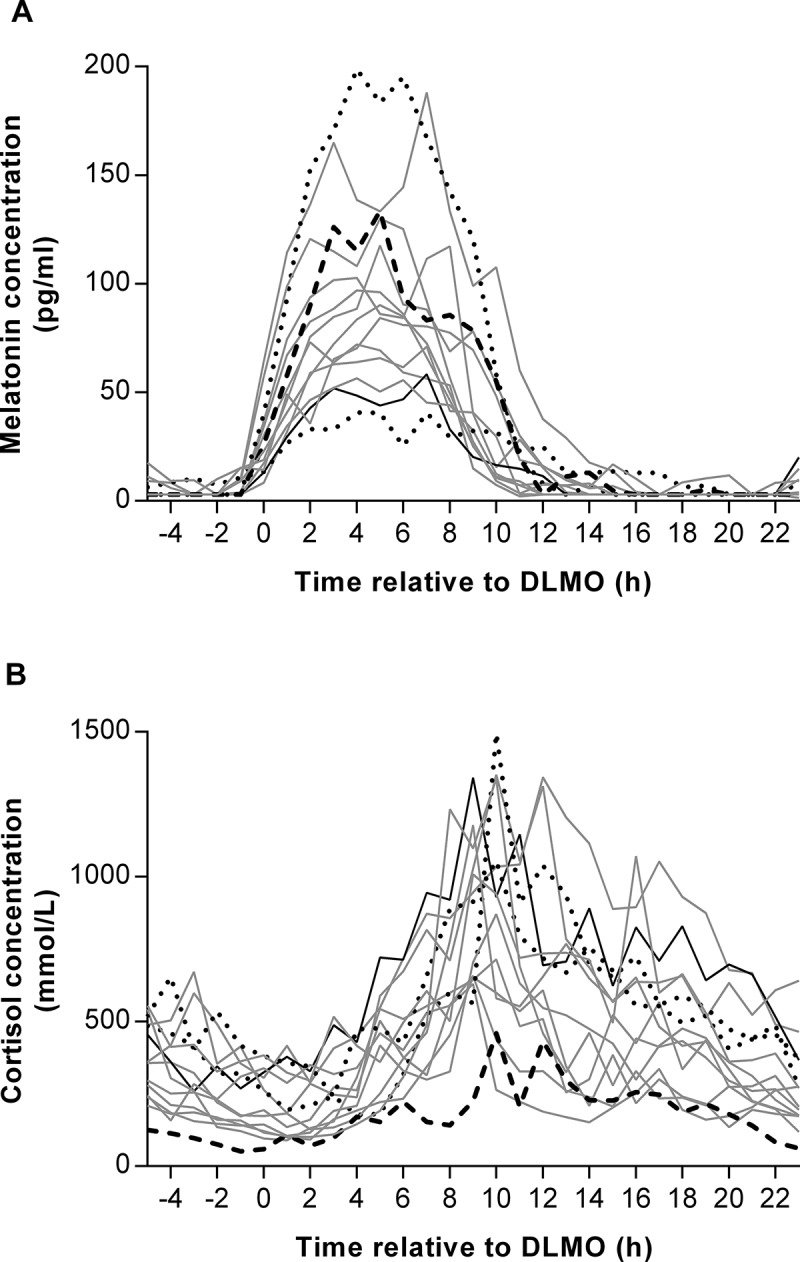
Individual plots of plasma melatonin (**A**) and cortisol (**B**) concentrations measured at hourly intervals during a constant routine (CR) protocol in female participants according to hormone dose: 0 µg ethinylestradiol and 75 µg progestin (long-dashed line, *n* = 1), 30 µg ethinylestradiol and 150 µg progestin (solid grey line, *n* = 10), 30 µg ethinylestradiol and 3000 µg progestin (short-dashed line, *n* = 2), 35 µg ethinylestradiol and 250 µg progestin (solid black line, *n* = 1). Data are plotted relative to each person’s DLMO_25%_, designated to occur at 0 h.

To investigate the effects of exogenous hormone administration from OCP on plasma melatonin and cortisol, individual rhythms in female participants were plotted according to the OCP that they were taking (Figure 3). When the AUC for plasma melatonin was divided into quartiles (interquartile range: 666 to 1057 pg/ml.h), there was a trend for a negative association with ethinylestradiol dose, with the participant on the highest dose (35 µg/day) in the lowest quartile (511 pg/ml.h) and the participant on the lowest dose (0 µg/day) in the highest quartile (1172 pg/ml.h). By contrast, when the plasma cortisol AUCs were split into quartiles (interquartile range: 9775 to 17348 mmol/L.h), there was a positive association, with the participant on the highest ethinylestradiol dose in the upper quartile (19831 mmol/L.h), and the participant on the lowest dose in the lowest quartile (5345 mmol/L.h). There were no such trends present when progestin dose was analysed in a similar way.

## Discussion

This study set out to assess whether sex differences in the circadian rhythms of melatonin, cortisol and their urinary excretion products were observed in young, healthy participants under highly controlled, CR conditions. Although we did not observe any differences in timing (melatonin onset (DLMO_25%_), offset (DLMOff_25%_) and acrophase), we did observe significant sex differences in the circulating and excreted levels of melatonin and cortisol over the 32-hour CR. The elevated levels of both plasma melatonin and cortisol production observed in females compared to males were not reproduced in the urinary excretion of aMT6s and cortisol. In fact, while there were no differences in aMT6s production, cortisol excretion was significantly higher in males, contradicting the plasma results. The observed sex differences may be endogenous and/or OCP-driven; preliminary analysis of OCP formulation showed a potential effect of ethinylestradiol dose on melatonin and cortisol production.

The current data demonstrated no difference in circadian phase timing between men and women. This is in agreement with a previous report (Burgess and Fogg, 2008) but not others (Burgess and Eastman, 2005; Cain et al., 2010; Mongrain et al., 2004; Van Reen et al., 2013). It is unclear why these inconsistencies in the data exist, although some of it may be attributable to methodological differences. One reason males and females exhibited a similar DLMO in the Burgess and Fogg (2008) study may have been that the data were pooled from several studies with different habituation periods to the allocated sleep schedules (from three to 15 days) (Burgess and Fogg, 2008). In addition, bedtimes for men and women were not reported and variable bedtimes could have masked any underlying gender differences in endogenous timing, a possibility eliminated by the matching of participants by sleep schedule in other studies (Cain et al., 2010; Van Reen et al., 2013). The strict recruitment of participants in our study, with a tightly regulated habitual bedtime within a three-hour window, time in bed of eight hours, and exclusion of extreme ‘early’ or ‘late’ chronotypes, meant the male and female participants did not differ significantly in their habitual bed and wake times, but this may also have resulted in a narrow spread of data and prevented differences in phase from being detected. Although not statistically significant, the comparison of timing of the plasma cortisol acrophase in our data approached significance (*p* = 0.079), with women tending to have earlier cortisol acrophase times than men. Previous studies have found women to be more ‘morning type’ than males, have earlier rhythms of core clock gene expression and exhibit a shorter circadian period (Adan and Natale, 2002; Duffy et al., 2011; Lim et al., 2013). Therefore, the non-significant trend in our study supports these previous data by suggesting that women tend to have an earlier phase than men, despite selecting similar bed and wake times.

Although no differences were detected in the timing of melatonin rhythms (onset, offset and acrophase), there were clear differences in the amount of melatonin produced by male and female participants, in agreement with a previous study (Cain et al., 2010). The reason for elevated plasma melatonin production in females is not completely understood. One plausible explanation may be differences in melatonin synthesis caused by sex hormones; the presence of melatonin receptors in human reproductive organs and evidence of reciprocal sex hormone receptors in the pineal gland suggest a regulatory relationship between sex hormones and melatonin (Ekmekcioglu, 2006; Luboshitzky et al., 1997). In addition, animal studies demonstrate a stimulatory effect of estradiol and an inhibitory effect of testosterone on melatonin secretion from the pineal gland (Cardinali et al., 1987). It is plausible that exogenous hormones from the OCPs used by females in our study may have contributed toward our findings. Although, in contrast to the animal data, our preliminary analysis suggested that increasing ethinylestradiol dose may be associated with lower plasma melatonin levels within the female group, the lack of a non-OCP taking control group makes drawing definitive conclusions from our results difficult. Previous research has reported an increase in melatonin production in OCP users compared to non-users (Burgess and Fogg, 2008; Kostoglou-Athanassiou et al., 1998), which could imply that the differences in melatonin we noted between sexes were caused by exogenous hormones from the OCP in females. However, given that the magnitude of differences in total and peak melatonin between men and women in our study are almost identical to a previous report where females were not taking the OCP (Cain et al., 2010), it is unlikely that contraceptive hormones alone account for the differences observed between sexes. However, despite being unable to pinpoint the cause of the higher plasma levels of melatonin (OCP and/or sex difference), the physiological implications of elevated melatonin levels in females deserve further study.

In contrast to plasma levels of melatonin, urinary levels of aMT6s did not differ between men and women. Although individual production of melatonin and excretion of aMT6s was highly correlated, a difference between sexes was apparent when mean levels for each group were compared. This was not a result of increased urine production in males as this was remarkably similar between groups (data not shown). Likewise, clearance rate of melatonin did not differ significantly between groups. Melatonin is metabolised by the liver, where it is hydroxylated to 6-hydroxymelatonin primarily by CYP1A2, before being conjugated with sulfate to form aMT6s and excreted in the urine (Ma et al., 2005). A recent study which examined melatonin sulfation in a number of human tissue samples found that women either metabolised melatonin at a higher or equal rate to men, depending on the tissue examined (Tian et al., 2015). This supports previous findings in a multinational study which showed a higher excretion of melatonin in females compared to males (Wetterberg et al., 1999). However, in this study, aMT6s was not measured, only a single sample was taken once a month over 12-16 months, and it is not clear how many women were on the OCP, making a comparison between these results and ours difficult. The OCP may have influenced melatonin metabolism and excretion in the females in the present study. In the presence of ethinylestradiol, the activity of hydroxylation enzymes in humans and sulfation of melatonin and 6-hydroxymelatonin in human liver tissue has been found to decrease (Papagiannidou et al., 2014; Shelepova et al., 2005), suggesting inhibition of melatonin metabolism in females taking OCPs. Thus, in OCP-users, a possible concomitant elevation in melatonin synthesis, as evidenced by human and animal studies (Burgess and Fogg, 2008; Cardinali et al., 1987; Kostoglou-Athanassiou et al., 1998) and inhibition of melatonin metabolism by ethinylestradiol may account for elevated plasma melatonin. Inhibited melatonin metabolism may also explain why the high plasma levels observed in the females in the current study do not translate into high aMT6s levels in the urine.

Similar to melatonin, plasma cortisol levels and amplitude were significantly higher in females. Previous studies have yielded inconsistent results when assessing sex differences in cortisol levels (Kudielka et al., 2004; Larsson et al., 2009; Seeman et al., 2001). This may be due to the use of only one or two time points to measure cortisol when the entire, 24-hour, profile needs to be captured in order to make accurate assessments of circadian phase and amplitude. One previous study which measured cortisol across 24 hours, however, showed that total cortisol levels were higher in males than non-OCP-using females (Van Cauter et al., 1996). The discrepancy between these results and the current findings could be a result of females in our study taking the OCP. It is well-established that exogenous estrogen increases corticosteroid binding globulin (CBG) and, therefore, increases total cortisol levels in the blood (Simunkova et al., 2008). Some additional reports suggest that free cortisol may also increase with OCP use, likely through increased basal activation of the hypothalamic-pituitary-adrenal-axis (Boisseau et al., 2013; Meulenberg et al., 1987). Indeed, the one female participant in our analysis who was not taking estrogen but was taking a progesterone-only pill had cortisol levels similar to that of the male participants. The difference in OCP formulations also likely explains the positive trend we observed between ethinylestradiol dose and cortisol levels (Brien, 1975; Burke, 1969).

The finding of lower urinary cortisol in females compared to males, supported by a reduced excretion rate in women, is in line with previous reports; both free cortisol and total cortisol metabolites have been found to be elevated in males compared to females, despite there being no differences in urinary volume (Lamb et al., 1994; Shamim et al., 2000). Our data are somewhat difficult to interpret since total cortisol was measured in the plasma and free cortisol in the urine. Cortisol is metabolised primarily in the liver and kidney, with a number of metabolites formed depending on the enzymatic pathway entered (Finken et al., 1999), which, along with free cortisol, can then be excreted in the urine. As well as increasing levels of bound and free cortisol (Boisseau et al., 2013; Meulenberg et al., 1987; Simunkova et al., 2008), estrogen reduces the ability of the liver to metabolise cortisol (Sandberg et al., 1969). The combination of an increase in plasma bound, inactive, cortisol and a reduced rate of metabolism is likely to be the reason that although females had significantly higher level of circulating cortisol, they excreted significantly less cortisol compared to males. A previous study which compared total plasma cortisol to urinary cortisol expressed relative to creatinine in females not taking the OCP (Jung et al., 2014) found a moderate correlation between these measures (*r* = 0.61), similar to the magnitude, albeit non-significant, found in the males in our study. Another possibility is that females diverted more free cortisol into metabolic pathways; previous studies have noted a difference in the activities of enzymes related to cortisol metabolism in males and females (Finken et al., 1999; Raven and Taylor, 1996). However, it is not possible to determine the contribution of these different enzyme activities to the results obtained in the present study.

One strength of this study was the strict gold standard CR conditions used to investigate the endogenous clock-driven component of the melatonin and cortisol rhythms. In addition, we have been able to comprehensively assess these rhythms with the use of plasma and urine samples collected over an extended period of 32 hours. This is the first time, to our knowledge, that plasma and urine have been measured in the same individuals and during the same CR protocol. Our results therefore offer new insight into potential differences in the regulation, production and metabolism of melatonin and cortisol between males and females. The comparatively low participant numbers taking different OCPs, however, has prohibited us from carrying out statistical analysis on the different OCP data. Further analysis comparing women taking and not taking the OCP will help to identify sex effects resulting from exogenous hormone administration alone and those that remain due to differences in endogenously produced sex hormones.

In conclusion, no differences in circadian timing were found between males and females during a CR protocol. Significantly elevated levels of plasma melatonin and cortisol were observed in females, and significantly elevated urinary cortisol was detected in males. It was not possible to distinguish the contribution of innate sex differences and the use of the OCP toward these results. The loss of the observed plasma differences in the urinary data suggest that aMT6s and urinary free cortisol may not be accurate proxy markers for plasma melatonin and cortisol production, particularly in participants taking exogenous contraceptive hormones.

## Acknowledgements

The authors thank the Surrey Clinical Research Centre medical and clinical research teams for their help with the study and Cheryl Isherwood for designing the isocaloric snacks for the constant routine protocol. Drs. Florence Raynaud (Institute of Cancer Research, London) and Alfred Thumser (University of Surrey) helped to design the experiment. Assay reagents were obtained from Stockgrand Ltd. This work was supported by a grant from the UK Biotechnology and Biological Sciences Research Council (BB/I019405/1). D.J.S. is a Royal Society Wolfson Research Merit Award holder.

## Declaration of Interest

D.J.S. and B.M. are co-Directors of Stockgrand Ltd. V.L.R. is a scientific advisor to Lumie.
